# Comprehensive Glycomics of a Multistep Human Brain Tumor Model Reveals Specific Glycosylation Patterns Related to Malignancy

**DOI:** 10.1371/journal.pone.0128300

**Published:** 2015-07-01

**Authors:** Jun-ichi Furukawa, Masumi Tsuda, Kazue Okada, Taichi Kimura, Jinhua Piao, Shinya Tanaka, Yasuro Shinohara

**Affiliations:** 1 Laboratory of Medical and Functional Glycomics, Graduate School of Advanced Life Science, and Frontier Research Center for Post-Genome Science and Technology, Hokkaido University, Sapporo, Japan; 2 Department of Cancer Pathology, Hokkaido University Graduate School of Medicine, Sapporo, Japan; Swiss Institute of Bioinformatics, SWITZERLAND

## Abstract

Cancer cells frequently express glycans at different levels and/or with fundamentally different structures from those expressed by normal cells, and therefore elucidation and manipulation of these glycosylations may provide a beneficial approach to cancer therapy. However, the relationship between altered glycosylation and causal genetic alteration(s) is only partially understood. Here, we employed a unique approach that applies comprehensive glycomic analysis to a previously described multistep tumorigenesis model. Normal human astrocytes were transformed via the serial introduction of hTERT, SV40ER, H-RasV12, and myrAKT, thereby mimicking human brain tumor grades I-IV. More than 160 glycans derived from three major classes of cell surface glycoconjugates (N- and O-glycans on glycoproteins, and glycosphingolipids) were quantitatively explored, and specific glycosylation patterns related to malignancy were systematically identified. The sequential introduction of hTERT, SV40ER, H-RasV12, and myrAKT led to (i) temporal expression of pauci-mannose/mono-antennary type N-glycans and GD3 (hTERT); (ii) switching from ganglio- to globo-series glycosphingolipids and the appearance of Neu5Gc (hTERT and SV40ER); (iii) temporal expression of bisecting GlcNAc residues, α2,6-sialylation, and stage-specific embryonic antigen-4, accompanied by suppression of core 2 O-glycan biosynthesis (hTERT, SV40ER and Ras); and (iv) increased expression of (neo)lacto-series glycosphingolipids and fucosylated N-glycans (hTERT, SV40ER, Ras and AKT). These sequential and transient glycomic alterations may be useful for tumor grade diagnosis and tumor prognosis, and also for the prediction of treatment response.

## Introduction

Glycosylation of cellular proteins and lipids is essential for the maintenance of the normal physiological state of mammalian cells, while dysregulated glycosylation is closely related to various human diseases, including most life-threatening cancers [1, 2]. Indeed, the majority of Federal Drug Administration (FDA)-approved tumor markers, which are clinically utilized worldwide, are either glycans or glycoproteins [3]. Drug-resistant cancer cells typically exhibit stem-like properties and express distinctive glycosylated antigens, such as stage-specific embryonic antigen (SSEA) 4 and cluster of differentiation (CD) 133 [4, 5]. These glycosylated molecules are the preferred molecular targets for cancer cell eradication. Thus, elucidation and manipulation of glycosylation will form a versatile means of advancing human cancer therapy. In cancer, a near-universal feature of tumor cells is an altered glycosylation pattern relative to the normal tissue from which the atypical cells were initially derived [6]. Nevertheless, it remains unclear at which point in the tumorigenic process the aberrant glycoforms are acquired [7].

In the case of brain tumors, several glycomic alterations have been also previously reported. Brain tumors, especially malignant gliomas, are the most aggressive tumors in humans. Despite recent advances in therapeutic strategies, no curative treatment has been established for malignant gliomas [8]. Gliomas are categorized as grade I–IV according to the World Health Organization (WHO) classification of central nervous system tumors. Grade I and II tumors are considered benign, while grade III tumors and grade IV glioblastomas are malignant [9]. Because some low-grade gliomas progress to malignant tumors within a decade, early detection of the tumors in the subclinical state is critical to therapeutic success. Unfortunately, however, early identification of WHO grade I or II gliomas is difficult due to the lack of reliable biomarker(s) and the paucity of sensitive and specific detection methods. Previously reported glycomic alterations include up-regulated biosynthesis of globotriceramide (Gb3) and globotetraosylceramide (Gb4), neolactotetraosylceramide (nLc4), 3’/6’-sialyl-nLc4, and stage-specific embryonic antigen (SSEA)-4 as well as down-regulation of various gangliosides (e.g., asialo-GM1, GD1a, and GD1b) [10, 11]. Furthermore, increased expression of N-glycans containing bisecting GlcNAc [12], β1,6-linked GlcNAc [13], and fucosylated residues also correlates with the development of various brain tumors [14], as does the relative down-regulation of α2,6-linked sialic acid(s) [15]. In addition, focused microarray analysis of glyco-gene expression in human glioblastomas identified various genes that are more highly expressed in gliomas relative to normal brain, including O-fucosyltransferase 1 (POFUT1) and chitinase 3-like 1 (CHI3L1) [16]. Unfortunately, however, none of these alterations are clinically utilized worldwide for the diagnosis of brain tumors.

The above-mentioned findings about glycomic alteration were largely uncovered by comparing the expression levels of specific glycans between normal and cancerous tissues using histochemical approaches. However, considering that tumors develop and progress through a variety of mechanistic pathways, the identification of novel tumor biomarker candidates based on clinical samples alone may have limited success. Therefore, a genetically homogeneous brain tumor model would provide an important alternative to clinical samples to dampen the noise from genetic and environmental variations.

The multistep theory of tumorigenesis (tumor initiation, promotion, and progression) is now widely accepted. Hahn et al. first reported that the introduction of telomerase (by way of human telomerase reverse transcriptase, hTERT), simian virus 40 (SV40) T-antigen (to disrupt expression of the p53 and retinoblastoma (Rb) tumor suppressor proteins), and oncogenic RasV12 was sufficient to induce the transformation of human primary fibroblasts [17]. In the case of human gliomas, primary glioblastomas initially arise as grade IV tumors and possess *EGFR* amplification, *p16* ^*INK4A*^ deletion, and *PTEN* mutations. On the other hand, accumulation of mutations in the promoter regions of *hTERT*, and in the specific exon of *IDH1* (encoding isocitrate dehydrogenase 1), and of *p53* are reported to be associated with secondary glioblastomas, which initially arise as benign tumors but progress to an aggressive phenotype with multiple recurrences [18, 19]. Based on these earlier studies, we recently established a multistep model of human brain tumorigenesis through the serial introduction of hTERT, SV40 early region (ER) inducing large T- and small t-antigens, RasV12, and myrAKT (a constitutively active form of AKT with a myristoylation signal sequence) into primary normal human astrocytes (NHAs) [20].

The present investigation employed serially transformed NHA cells and recently established state-of-the-art quantitative analytical etchniques [21] to systematically assess alterations in the three major classes of cell surface glycans/glycoconjugates: the N- and O-glycans on glycoproteins, and the glycosphingolipids (GSLs). Consequently, a comprehensive view of the relationship between multiple successive genetic alterations and the resultant glycomic profiles was disclosed, permitting an establishment of the causal association between altered glycosylation and deregulated cellular pathways.

## Materials and Methods

### Cell culture

NHA cells (TaKaRa, Japan) were cultured in astrocyte growth medium (Lonza, Walkersville, MD, USA). To create an experimental human glioma model, NHA cells were introduced with genes for *hTERT* (T), *SV40ER* (S), *H-RasV12* (R), and *myrAKT* (A), as previously reported (20). The cells were maintained in Dulbecco’s modified Eagle’s medium (Sigma-Aldrich, St. Louis, MO, USA) supplemented with 10% fetal bovine serum (FBS) (Cansera, Ontario, Canada). After washing the culture dish with cold phosphate buffered saline (PBS), the cells were scraped into cold PBS containing 10 mM EDTA and collected by centrifugation at 1,000 × *g* for 3 min. The pellet was washed four times with phosphate-buffered saline (PBS) to remove fetal bovine serum (FBS), thereby minimizing contamination from FBS-derived glycoproteins. The cells were stored at −30°C as pellets (1 × 10^6^ cells/pellet). The total cellular protein concentration was determined by using the bicinchoninic acid (BCA) method (Pierce Biotechnology, Rockford, IL, USA).

### Immunoblotting

Cells were washed twice with cold PBS and lysed in a buffer containing 0.5% NP-40, 10 mM Tris-HCl (pH 7.4), 150 mM NaCl, 1 mM EDTA, 50 mM NaF, 1 mM PMSF, and 1 mM Na_3_VO_4_. The lysate was clarified by centrifugation at 15,000 rpm for 10 min, and the supernatants were subjected to SDS-PAGE in 10–12% polyacrylamide gels. Separated proteins were transferred to polyvinylidene difluoride membranes and blocked with Tris-buffered saline (TBS) containing Tween-20 (TBS-T) and 5% skim-milk at room temperature for 1 h. The membranes were incubated with primary antibodies at 4°C overnight, washed with TBS-T, incubated with secondary antibodies, and washed three more times with TBS-T. Immunoreactive signals were then detected by using an enhanced chemiluminescence (ECL) kit (GE Healthcare, Buckinghamshire, UK) and an ImageQuant LAS4000 imaging system (GE Healthcare). Primary antibodies were as follows: anti-hTERT (Abcam, Cambridge, UK); anti-Akt and anti-phospho-Akt (S473) (Cell Signaling Technology, Beverly, MA, USA); anti-SV40 T-antigen (clone Ab-1 for large T-antigen and clone Ab-3 for small T-antigen) (Oncogene Research Products, Dublin, OH, USA); and anti-H-Ras (BD Transduction Laboratories, Lexington, KY, USA).

### Extraction of (glyco)proteins

For N-glycan analysis, (glyco)proteins were extracted as described [21]. Briefly, cell pellets consisting of 1 × 10^6^ cells were homogenized using an Ultrasonic Homogenizer (Taitec Corp., Saitama, Japan) in 100 mM Tris-acetate buffer (100 μL, pH 7.4) supplemented with 2% sodium dodecyl sulfate as a surfactant for the complete dissolution of cell pellets. Reductive alkylation of the cellular proteins was performed by the addition of 500 mM Tris(2-carboxyethyl)phosphine (Sigma-Aldrich) at room temperature for 60 min, followed by the addition of 200 mM iodoacetamide (Sigma-Aldrich) at room temperature for 30 min. After reductive alkylation, ethanol precipitation was carried out adding a 4-fold volume of cold ethanol and incubation for 3 h at −30^°^C. Supernatants and precipitated proteins were separated by centrifugation at 20,000 × *g* for 10 min at 4^°^C, and precipitates were again washed with cold ethanol. Collected precipitates containing glycoproteins/N-glycans were dried at 37^°^C for 10 min.

### Extraction of GSLs

The GSL extraction procedures were essentially the same as previously described [22]. Briefly, defatting was performed by adding a chloroform/methanol (C/M) solution (2/1, v/v; 450 μL) to the cell pellets, followed by sonication at room temperature as described above for the extraction of glycoproteins/N-glycans. Methanol (150 μL) was then added, yielding a solvent composition of C/M = 1/1 (v/v). Sonication was repeated in the same manner. Finally, methanol (300 μL) was added (C/M = 1/2, v/v), and sonication was repeated once again. The resulting extracts were subjected to centrifugation at 20,000 × *g* for 10 min, and the supernatants were subjected to GSL-glycan analysis. The supernatants containing crude cellular lipids were completely dried using a centrifugal evaporator and subjected to digestion with ceramidase.

### Release of N-glycans

Ethanol-precipitated proteins were dissolved in 100 mM ammonium bicarbonate and digested overnight at 37^°^C by the addition of trypsin (Sigma-Aldrich) and was subjected to reductive alkylation as described [23]. Following deglycosylation by overnight treatment with peptide N-glycanase F (PNGase F, 2 U) (Roche, Basel, Switzerland), the resulting samples were dried using a centrifugal evaporator and dissolved in deionized water.

### Release of glycans from GSLs

Crude cellular lipids were suspended in 50 mM Tris-HCl buffer, pH 5.5 (50 μL) containing 0.2% Triton X-100 (Sigma-Aldrich) as a surfactant, followed by the addition of *Rhodococcus* endoglycoceramidase (EGCase) I and II (25 mU of each) (Takara Bio Inc., Shiga, Japan) to release intact glycans from GSLs. Enzymatic digestion was performed at 37°C for 24 h. A mixture of EGCase I and II was employed to maximize the release of glycan moieties from the three major classes of GSLs (ganglio-, (neo)lacto- and globo-series GSLs). To distinguish GSL-glycans from contaminating free oligosaccharides, crude cellular lipids were also suspended in Tris-HCl buffer/Triton X-100 in the absence of EGCases. The latter served as a negative control [22].

### RNA extraction and real-time PCR

Total RNA was isolated from the cells using the RNeasy Mini Kit (Qiagen Inc., Valencia, CA, USA) according to the manufacturer’s instructions. First-strand cDNA was synthesized from 0.5 μg of total RNA using SuperScript VILO reverse transcriptase (Invitrogen, Carlsbad, CA, USA). Quantitative real-time PCR (qPCR) was performed using a StepOnePlus real-time PCR system (Applied Biosystems, Foster City, CA, USA). Expression levels of the target genes were normalized to the level of glyceraldehyde 3-phosphate dehydrogenase (GAPDH).

### Glycoblotting

N-glycans and GSL-glycans were subjected to glycoblotting, as previously described [24]. In brief, the sample solution (20–50 μL) containing a known amount of internal standard was directly applied to the well of a filter plate (MultiScreen Solvinert 0.45 μm Low-Binding Hydrophilic PTFE, Millipore, Billerica, MA) containing BlotGlyco beads (5 mg) (Sumitomo Bakelite Co. Ltd., Tokyo, Japan). Glycans were captured in 2% acetic acid in acetonitrile (450 μL) and incubated at 80°C for 45 min. On-bead acetyl capping of unreacted hydrazide groups was performed by using 10% acetic anhydride in methanol for 30 min at room temperature. Next, on-bead methyl esterification of the carboxyl groups in glycan-derived sialic acid was carried out by incubation with 150 mM 3-methyl-1-p-tolyltriazene in dioxane at 60°C. The trapped and esterified glycans on the beads were subjected to transiminization by incubation with a mixture of 2% acetic acid in acetonitrile (180 μL) and 20 mM *N* ^α^-((aminooxy)acetyl)tryptophanylarginine methyl ester (aoWR, 20 μL), a dipeptidic aminooxy compound that was synthesized as described [25]. The aoWR-labeled glycans were recovered in distilled water (100 μL), and the collected solution was purified by using a hydrophilic interaction liquid chromatography (HILIC) purification plate (MassPrep HILIC μElution plate; Waters, Milford, MA, USA) to remove the excess aoWR. To concentrate the aoWR-labeled glycans, the purified solution was desiccated by using a rotational evaporator and subsequently dissolved in distilled water (10 μL).

### Cellular O-glycomic analysis by β-elimination in the presence of pyrazolone analogs (BEP)

The extracted cellular (glyco)proteins were subjected to BEP as previously described [26], with some modifications. Briefly, the extracted (glyco)proteins from the cell pellets were subjected to the same procedure as described above for N-glycan preparation. Collected proteins were concentrated by using an Amicon Ultra Centrifugal Filter with a 30K cutoff (Millipore) and subjected to the BEP reaction. Next, PMP-labeled GN4 was added to the reaction mixture and the solution was neutralized with 1.0 M hydrochloric acid. Chloroform was added, and the mixture was shaken vigorously. The chloroform layer was discarded to remove excess reagents, and the resultant aqueous layer was subjected to purification on a graphitized carbon column and an Iatrobeads silica gel column (Shell-usa, Spotsylvania, VA, USA). More detailed technical procedure for cellular O-glycome will be reported elsewhere.

### MALDI-TOF/TOF (tandem MALDI-TOF) MS analysis

Purified N-glycans, O-glycans, and GSL-glycan solutions were mixed with 2,5-dihydrobenzoic acid solution (10 mg/mL in 30% acetonitrile) and subsequently subjected to MALDI-TOF MS analysis as previously described, with minor modifications. Briefly, all measurements were performed using an Ultraflex II TOF/TOF mass spectrometer equipped with a reflector and controlled by the FlexControl 3.0 software package (Bruker Daltonics GmbsH, Bremen, Germany) according to general protocols. All spectra were obtained in reflectron mode with an acceleration voltage of 25 kV, a reflector voltage of 26.3 kV, and a pulsed ion extraction of 160 ns in the positive ion mode. The spectra were the results of signal averaging of 1000–4000 laser shots. All peaks were picked using FlexAnalysis 3.0 with the SNAP algorithm that fits isotopic patterns to the matching experimental data. The external calibration was performed using the mixture of Man3-Man9 N-glycans and internal standard, Neu5Ac_2_Gal_2_GlcNAc_2_ + Man_3_GlcNAc_1_(A2GN1). In TOF/TOF mode measurement for fragment ion analysis, precursor ions were accelerated to 8 kV and selected in a timed-ion gate. Fragment ions generated by laser-induced decomposition of the precursor were further accelerated by 19 kV in the LIFT cell. Fragment masses were analyzed after passing through the ion reflector.

### Peak lists, composition estimation and quantitation

Mass spectrometry (MS) peak lists were generated by using FlexAnalysis 3.0 (peak finder settings: signal-to-noise (S/N) threshold: 1.5) without further smoothing or spectrum processing. The glycan compositions were manually determined by conducting a database search (i.e. a compositional search of the UniCarbKB database (http://www.unicarbkb.org/query) for N- and O-glycans, and of the SphinGOMAP database (http://www.sphingomap.org/) for GSL-glycans). All previously documented GSL glycans in SphinGOMAP database were extracted and compiled as an in-house database to allow searching for glycans by m/z value and/or composition. The parameters for searching were fixed modifications of aoWR at reducing terminal, variable modifications of methyl esterification of sialic acid(s), glycan mass tolerance: ± 0.5 Da, glycan charge of 1+ (due to the high proton affinity of aoWR). Each peak was assigned a putative topology, based on its m/z value and plausible biosynthetic pathway. Some of the glycan structures were further examined by tandem time-of-flight/time-of-flight (TOF/TOF) MS and enzymatic digestion. Absolute quantification was performed by comparative analyses between the areas of the MS signals derived from each glycan and a known amount of A2GN1 (in the cases of N- and GSL-glycans) and GN4 (in the case of O-glycans). The glycan expression amounts were normalized to an equivalent amount of total protein amount (100 μg).

### Exoglycosidase-assisted structural analysis

The aoWR-labeled N-glycans were digested with *Salmonella typhimurium* neuraminidase to estimate the relative amounts of α2,3- and α2,6-linked sialic acids. Recombinant α2,3-neuraminidase (50 U) derived from *S*. *typhimurium* (New England BioLabs Inc., Beverly, MA, USA) was added to 10 μL of final solution obtained from the glycoblotting procedure. The solution was incubated at 37°C for 20 h and an aliquot was directly mixed with 2,5-dihydrobenzoic acid (DHB) and subjected to MS analysis without further purification. Next, aoWR-labeled GSL-glycans were digested with β1–3,6-galactosidase to distinguish between the isomer structures possessing different linking modes of galactose residue(s) (e.g., to distinguish Gb4 from (n)Lc4, and GM1/GM1α (Gg) from sialyl- (n)Lc). Recombinant β1–3,6-galactosidase (100 mU) derived from *Xanthomonas manihotis* (Calbiochem, Darmstadt, Germany) was added to 12 μL of the final solution obtained from the glycoblotting procedure. The solution was incubated at 37°C for 3 h, and an aliquot was directly mixed with DHB and subjected to MS analysis without further purification. For O-glycans, the proteins/glycoproteins extracted from cell pellets were treated with a mixture of β1,3-galactosidase derived from *X*. *manihotis* and β1,4-galactosidase derived from *Streptococcus pneumoniae* (Calbiochem). Galactosidase-digested samples were then subjected to O-glycan analysis, as described.

### Cluster analysis

Cluster analyses were performed with software Cluster 3.0, developed by the Michael Eisen laboratory (http://www.eisenlab.org/eisen/) with a hierarchical clustering algorithm [27]. The calculated dendrogram and correlation matrix were visualized by using Java TreeView 1.1.6r4 software downloaded from the same site.

## Results

### Establishment of human glioma models, grades I-IV

**Fig 1:**
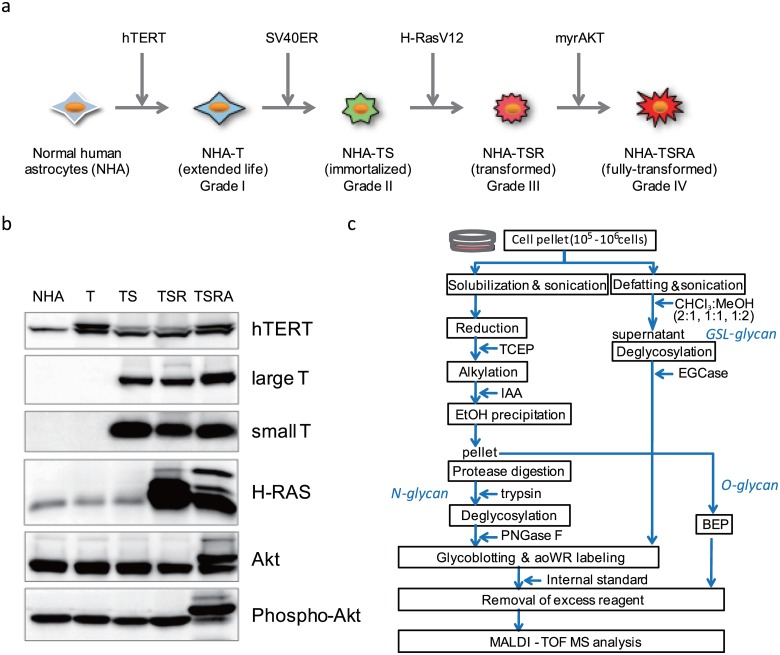
Model NHA cells and experimental design for the glycomic analysis. (a) Generation of immortalized and transformed human astrocytes. (b) Expression levels of specific defined elements (i.e., hTERT, SV40ER (both large T and small t), H-RasV12, and myrAKT) assessed by immunoblotting. (c) Protocols employed for glycomic analyses of N-, O- and GSL-glycans. For further details, see Methods.

We employed our original brain tumor model in which parental NHAs were transformed through the serial introduction of hTERT, SV40ER, H-RasV12, and myrAKT, and designated the transformed NHAs as NHA/T, NHA/TS, NHA/TSR, and NHA/TSRA cells, respectively. Although the NHA/TS cells exhibited a higher rate of cell growth than the parental NHAs, they failed to form colonies in soft agar, indicating their status as immortalized but not transformed. In our previous study, we found that NHA/TSR cells successfully formed a considerable number of colonies in soft agar, along with xenografted tumors in nude mice [20]. Intratumoral necrosis was displayed in the context of xenografted tumors formed by NHA/TSRA cells, with a higher proliferative index. Thus, NHA/TS cells appear to mimic benign grade II gliomas, whereas NHA/TSR and NHA/TSRA cells exhibit histopathological features of malignant grade III and IV gliomas, respectively [20] (Fig 1a).

The expression levels of hTERT, SV40 T- and t-antigens, H-RasV12, and myrAKT are shown in Fig 1b. H-Ras V12 expression seemed to be partially reduced in NHA/TSRA compared with NHA/TSR cells, probably due to a cellular protective effect against excessive oncogenic signaling generated by H-RasV12 and myrAKT.

The protocols employed for the glycomic analyses of N-, O- and GSL-glycans are summarized in Fig 1c. Cell pellets were divided into two groups: one for the analysis of N and O-glycans, and the other for the analysis of GSL-glycans. N- and GSL-glycans were released by digestion with PNGase F and EGCases I/II, respectively, and the released glycans were purified, methyl esterified on the carboxyl groups of sialic acid(s), and labeled with aoWR by a glycoblotting procedure. O-glycans were released and labeled by 1-phenyl-3-methyl-5-pyrazolone (PMP) via β-elimination in the presence of the pyrazolone analogue, BEP. Absolute quantitation of each glycan was performed by comparative analysis between the areas of the MS signal derived from each glycan and the internal standard. The basis of the quantitative analysis of glycans on MALDI-TOF MS relies on previous observations that oligosaccharides with masses of >~1,000 Da exhibit similar signal strengths, regardless of structure [24, 28], and that methyl esterification of sialic acid renders sialylated oligosaccharides chemically equivalent to neutral oligosaccharides, permitting simultaneous analysis of neutral and sialylated oligosaccharides [29, 30].

Some of the GSL- and O-glycans analyzed in this study have molecular masses of <1,000 Da, and therefore, the quantitative values obtained for these glycans may be underestimated, which is an issue to be addressed in the future. Although the current protocol for O-glycan analysis does not employ methyl esterification of sialic acid residue(s), we confirmed that the glycomic analysis of model glycoprotein was reproducible as shown in S1 Fig, and was comparable to that previously reported [31].

### Alterations in GSL-glycomics

#### A dynamic core structural switch occurs along with the transformation process

Up to 47 total GSL-glycans were detected in this study (S1–S5 Tables; S2 Fig). These glycans were assigned by referring to the SphinGOMAP database [32], and categorized into three series, (ganglio (Gg), globo (Gb), and (neo)lacto ((n)Lc)), according to their internal core carbohydrate sequence. We used tandem TOF/TOF MS and enzymatic digestion with exo-β-galactosidase to distinguish certain isomers from each other (e.g., Gb4 from (n)Lc4, and GM1/GM1α (Gg) from sialyl- (n)Lc). However, some isomers were very difficult to distinguish even after these experimental manipulations; thus, they were categorized as either “Gg or (n)Lc” or “Gb or (n)Lc” isomers.

**Fig 2:**
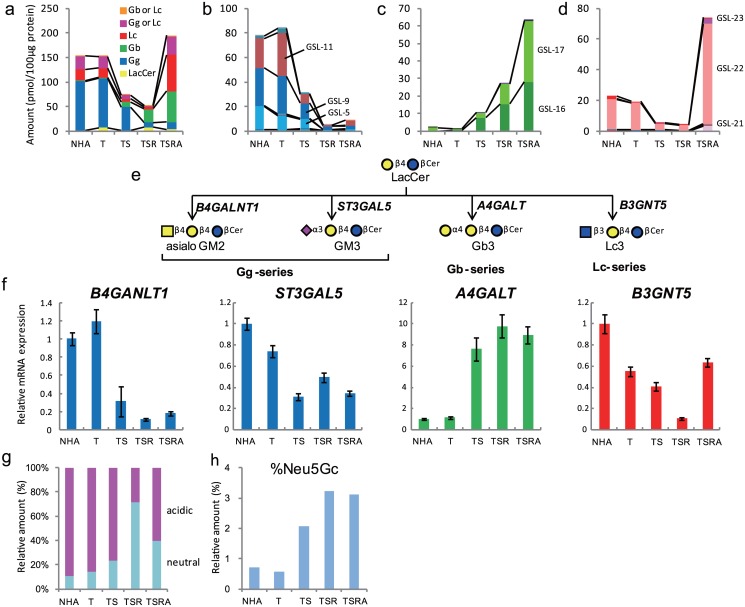
Cellular GSL-glycomes. Quantified GSL-glycans were stratified into ganglio (Gg), globo (Gb), (neo)lacto (n)Lc, “Gg or (n)Lc”, and “Gb or (n)Lc” groups according to glycan structure (a). Glycans classified into the Gg (b), Gb (c) and (n)Lc-series (d) were further compared according to the levels of constitutive GSL-glycans. SphinGOMAP (http://www.sphingomap.org/) online databases were used for structural estimation of GSL glycans, which are summarized in S1 Table. Biosynthetic pathways for the generation of Gg-, Gb-, and (n)Lc-series GSL-glycans (e) and real-time PCR analysis of glycosyltransferases involved in GSL-glycan biosynthesis (f). Each value represents the mean ± SD of three independent real-time PCR analyses. Comparison of the relative abundance of neutral and sialylated GSL-glycans (g), and comparison of relative levels of GSL-glycans with N-glycolylneuraminic acid incorporation among total sialylated GSL-glycans (h).

As shown in Fig 2a, the GSL-glycomic profile of each transformed cell type differed substantially. Introduction of hTERT resulted in few GSL-glycomic alterations, apart from an increase in the expression of lactosylceramide (LacCer) by ~7- fold. Subsequent introduction of SV40ER and H-RasV12 reduced the total amounts of GSL-glycans by 48 and 34%, respectively, while introduction of myrAKT markedly increased the total amounts of GSL-glycans. The Gg-series GSLs were the major components (> 65%) in parental NHA and NHA/T cells, while their expression decreased sharply by the subsequent introduction of SV40ER, H-RasV12 and myrAKT (Fig 2b). By contrast, although the Gb-series GSLs were scarce in NHA and NHA/T cells (< 2% of all GSL-glycans), their levels increased exponentially upon the sequential introduction of SV40ER, H-RasV12 and myrAKT (Fig 2c). The total content of (n)Lc-series GSLs was reduced to 30% of the level in parental NHA cells by the introduction of SV40ER. The (n)Lc-series GSLs were maintained at low levels after the ensuing introduction of H-RasV12, but their expression levels were drastically increased (> 16-fold) by the introduction of myrAKT (Fig 2d).

The common precursor LacCer is converted into Gg-, Gb-, and (n)Lc-series GSLs by GM3 synthases (encoded by *B4GALNT1 and ST3GAL5*), Gb3Cer synthase (encoded by *A4GALT*), and Lc3Cer synthase (encoded by *B3GNT5*), respectively (Fig 2e). As shown in Fig 2f, *B4GALNT1* and *ST3GAL5* were down-regulated to 27 and 42% of control levels, respectively, upon introduction of SV40ER, while *A4GALT* was up-regulated by 6.8-fold. These observations can readily explain the switch of core GSL structures from Gg- to Gb-series glycans. *B3GNT5* was gradually down-regulated by the successive introduction of hTERT, SV40ER, and H-RasV12, and markedly increased upon introduction of myrAKT, which is also in good agreement with the glycomic observations.

Overall sialylation of GSL-glycans is summarized in Fig 2g. Neutral GSL-glycans accounted for ~10–23% of total GSL-glycans in NHA, NHA/T, and NHA/TS cells, while the percentage climbed to ~71% in NHA/TSR cells and declined to ~40% in NHA/TSRA cells. The relative percentage of N-glycolylneuraminic acid (Neu5Gc, a non-human sialic acid)-carrying glycans among total sialylated GSL-glycans was < 1% in NHA and NHA/T cells, but increased to 2.3, 3.6, and 4.4% in NHA/TS, NHA/TSR, and NHA/TSRA cells, respectively (Fig 2h).

#### Identification of malignancy-related GSL-glycans

**Fig 3:**
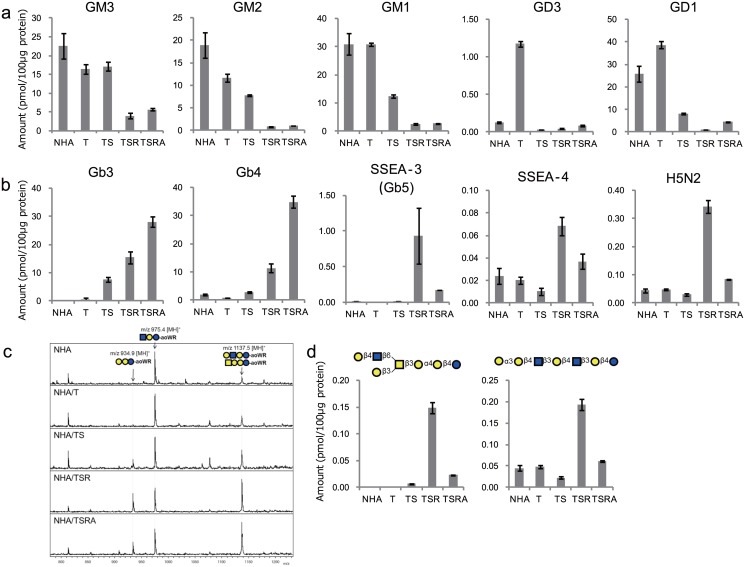
Expression profiles of representative GSL-glycans. (a) Gg-series glycans. (b) Gb-series glycnas. (c) MALDI-TOF/TOF MS spectra of GSL-glycans with a (Hex)5(HexNAc)2 composition. In theory, signature fragmentation peaks at m/z 934.9 and 975.4 are specifically generated from Gb- and (n)Lc-series GSL-glycans, respectively. (d) Expression profiles of Gb-series (Hex)5(HexNAc)2 glycan (left) and (n)Lc-series (Hex)5(HexNAc)2 glycans (left). The glycan amounts in (d) were calculated from the area of the signature fragmentation peaks (m/z 934.9 and 975.4) shown in (c). SphinGOMAP (http://www.sphingomap.org/) online databases were used for the structural estimation of the GSL glycans. Each value represents the mean ± the standard deviation (SD) of three independent MALDI-TOF MS-derived analyses.

Expression profiles of individual Gg- and Gb-series glycans are shown in Fig 3. As with the total Gg-series expression profile, GM1, GM2, GM3 and GD1 content decreased sharply by the introduction of SV40ER and H-RasV12. Moreover, a specific and transient increase (~16-fold) was observed in the expression of GD3 upon the introduction of hTERT (Fig 3a).

Regarding Gb-series glycans, Gb3 and Gb4 are the major species of Gb-series GSLs, and were increased exponentially upon successive introduction of SV40ER, H-RasV12 and myrAKT (Fig 3b). On the other hand, SSEA-4, a recently identified biomarker for high-grade astrocytomas, as well as SSEA-3, the precursor of SSEA-4, were both specifically amplified upon the introduction of H-RasV12. Intriguingly, we identified a glycan comprising (Hex)_5_(HexNAc)_2,_ which showed an expression profile similar to that of SSEA-4 (Fig 3b). According to the SphinGOMAP database, there are two possible structures: Gb-type Galβ1,4GlcNAcβ1,6(Galβ1,3)GalNAcβ1,3Galα1,4Galβ1,4Glc, and nLc-type Galα1,3Galβ1,4GlcNAcβ1,3Galβ1,4GlcNAcβ1,3Galβ1,4Glc. MS/MS analysis indicated that the latter was the only species in NHA and NHA/T cells, because the signature fragmentation peak (m/z 934.9) for Gb-series glycans was not detected. On the other hand, this signature peak was clearly observed in NHA/TS cells, and markedly increased in NHA/TSR cells (Fig 3c). We found that Gb-type (Hex)_5_(HexNAc)_2_ GSL was more specific for H-RasV12 introduction than SSEA-4 (Fig 3d).

Up to 19 (n)Lc-series GSL-glycans were detected in total, and sialyl-(n)Lc4 was the major species (S3a Fig). (n)Lc5, (n)Lc6, and their extended and/or branched polylactosamine (polyLacNAc) counterparts (with or without sialylation) were also detected in parental NHA cells, but only a few fucosylated glycans were observed in these cells (~0.01% of total GSL-glycans) (S3b Fig).

Few alterations occurred upon the introduction of hTERT, except that the inherently low numbers of fucosylated glycans were totally lost. Introduction of SV40ER not only caused a marked reduction in the total content of (n)Lc-series GSLs, but also many qualitative alterations, including the emergence of various fucosylated glycans (comprising ~0.7% of total GSL-glycans). H-RasV12 introduction reduced the levels of extended and/or branched polyLacNAc species, whereas myrAKT presentation again increased the extension of polyLacNAcs and/or branching GSLs, and markedly increased fucosylation (~2% of total GSLs) (S3c, S4 Figs).

### Alterations in N-glycomics

#### Expression of pauci-mannose type glycans specifically increases upon introduction of hTERT

**Fig 4:**
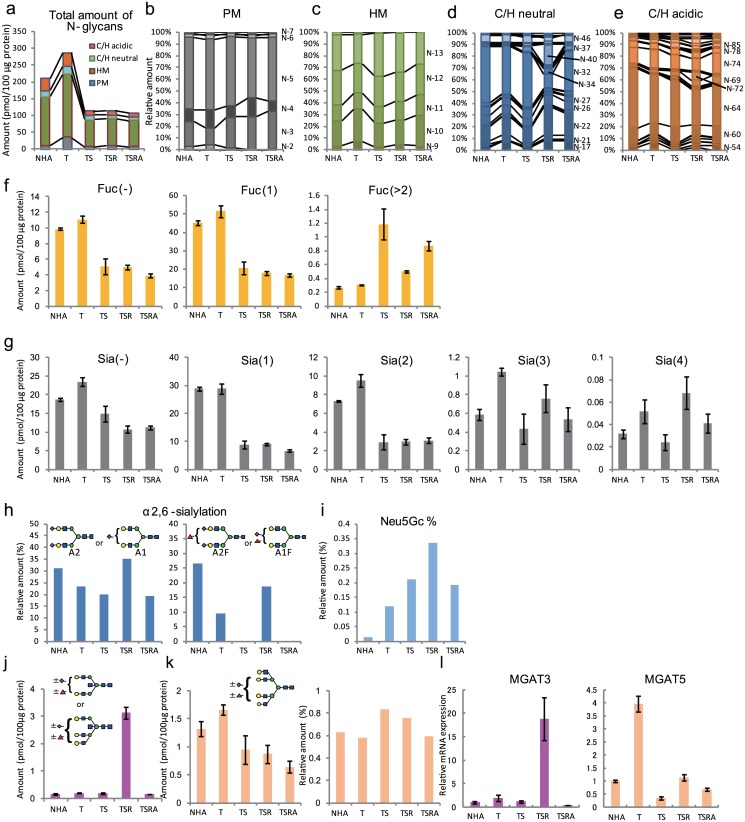
Cellular N-glycomes. Quantified N-glycans were structurally classified as PM-, HM-, and C/H-type glycans (a). PM- (b), HM- (c), C/H neutral- (d), and C/H acidic-type glycans (e) were further compared according to composition. Glycan classifications were performed based on the estimated glycan structures and compositions shown in S6–S10 Tables. Fucosylation and sialylation status of C/H-type N-glycans (f and g). (h) Proportion of α2,6 sialic acids among total sialic acids with A2 and A1 (left) and A2F and A1F (right). (i) Relative levels of N-glycans showing N-glycolylneuraminic acid incorporation among total sialylated N-glycans. Expression profiles of glycans with bisecting GlcNAc residues (j) and tetra-antennary glycans (k). (j) Sum of the expression levels of all glycans having (Hex)_2_(HexNAc)_3_ or (Hex)_3_(HexNAc)_4_ substructures were compared among NHA, NHA/T, NHA/TS, NHA/TSR, and NHA/TSRA cells. (k) Sum of the expression levels of all glycans having (Hex)_4_(HexNAc)_4_ as a substructure was compared among the different cell types. Left; absolute amounts, right: relative abundance of tetra-antennary glycans among C/H-type glycans. (l) Real-time PCR analysis of *MGAT3* (left) and *MGAT5* (right) expression levels. Each value represents the mean ± the standard deviation (SD) of three independent MALDI-TOF MS (f, g, j, and k) or real-time PCR analyses (l).

We quantitatively analyzed ~97 total N-glycans and demonstrated qualitative alterations in glycan profile upon ectopic expression of the various genes employed in this study (S6–S10 Tables; S5 Fig). These glycans were structurally classified into pauci-mannose (PM), high-mannose (HM), and complex/hybrid (C/H) types. A portion of the C/H types were modified with sialic acid(s) to produce acidic glycans. HM-type glycans were the major species (~70%) in all NHA cells (Fig 4a)., followed by the C/H acidic- and C/H neutral-type glycans. Although the PM-type glycans were minor components in the parental NHA cells, they increased by ~3-fold upon introduction of hTERT. Further introduction of SV40ER reduced the total amount of N-glycans to ~40%, and the N-glycan levels remained relatively steady after the successive introduction of H-RasV12 and myrAKT. These qualitative alterations in glycan composition were not particularly profound in the case of the PM-type and HM-type glycans (Fig 4b and 4c). However, distinct qualitative alterations were observed for the C/H-type glycans (Fig 4d and 4e), as described below.

#### Fucosylation and sialylation show unique expression patterns throughout the transformation process

The expression patterns of non-fucosylated and mono-fucosylated N-glycans showed a clear declining trend after the introduction of SV40ER, while those of N-glycans bearing multiple fucose residues were specifically increased upon the introduction of SV40ER, H-RasV12, and myrAKT (Fig 4f; S6 Fig). N-glycans showed various types of fucosylations, including terminal fucosylation via α1,2 linkages (encoded by *FUT1* and *FUT2*), α1,3/4 linkages (encoded by *FUT3*) and α1,3 linkages (encoded by *FUT4-7 and FUT9*), in addition to core α1,6-fucosylation (encoded by *FUT8*). The mRNA expression levels of assorted fucosyltransferase genes (*FUT1-9*) were detected by real-time PCR (S7 Fig). *FUT1* was gradually up-regulated by the successive introduction of hTERT, SV40ER, H-RasV12 and myrAKT, while *FUT3* and *FUT5* were up-regulated (~5.8- and ~3.3-fold) by the introduction of myrAKT. *FUT8* was specifically up-regulated by the introduction of H-RasV12.

The levels of mono- and di-sialylated N-glycans were reduced to only ~30% of the levels in the parental NHA cells by the introduction of SV40ER, while those of tri- and tetra-sialylated N-glycans remained fairly constant or even increased throughout the transformation process (Fig 4g). As shown in Fig 4h, left; S8 Fig), ~30% of sialic acids of biantennary N-glycans (A2 and A1) were α2,6-linked in NHA cells. The rates were reduced to 23% and 20% in NHA/T and NHA/TS cells, respectively, increased to 35% in NHA/TSR cells, and reduced to 19% in NHA/TSRA cells. These trends were also observed for the fucosylated analogs (A2F and A1F), but they were more pronounced; indeed, almost all A2F and A1F analogs were modified with α2,3-linked sialic acid residues in NHA/TS and NHA/TSRA cells (Fig 4h, right; S8 Fig).

Only 0.01% of N-glycans among the total sialylated N-glycans on NHA cells carried Neu5Gc. The Neu5Gc/total sialylated N-glycan ratio increased by 8.4-, 15.1-, 23.8-, and 13.6-fold in NHA/T, NHA/TS, NHA/TSR, and NHA/TSRA cells, respectively (Fig 4i).

#### Expression of glycans with bisecting GlcNAc and β1,6-branched GlcNAc residues is closely associated with the incorporation of H-RasV12 and hTERT, respectively

To evaluate any branch-specific expression trends, C/H-type N-glycans were classified into four groups depending on the number of HexNAc residues: mono-, bi-, tri-, and tetra-antennary glycans. The levels of mono-antennary glycans were specifically augmented by the introduction of hTERT (S9 Fig), whereas those of biantennary glycans were down-regulated by more than 2-fold after the introduction of SV40ER, and were then maintained at low levels even after the ensuing introduction of the activated form of H-Ras and AKT. The tri- and tetra-antennary glycans showed somewhat unique expression profiles, as discussed below.

Glycans categorized as tri- and tetra-antennary types include those with bisecting GlcNAc residues. Therefore, the expression levels were compared for glycans having (Hex)_2_(HexNAc)_3_ or (Hex)_3_(HexNAc)_4_ as substructures, because these are typical compositions for glycans bearing bisecting GlcNAc residues. Fig 4j and S9 Fig show that the content of such glycans increased markedly (> 15-fold) in malignant transformed NHA/TSR cells, but not in NHA/TS cells, indicating that their high expression was uniquely caused by the activation of Ras. On the other hand, we found that the introduction of hTERT moderately increased the expression of tetra-antennary glycans (the characteristic substructure for glycans containing β1,6-linked GlcNAcs) by ~26%, while the introduction of other genes instead decreased the expression of tetra-antennary glycans (Fig 4k, left). On the other hand, the relative abundance of tetra-antennary glycans among the C/H-type glycans increased in NHA/TS and NHA/TSR cells (Fig 4k, right).

We next explored the correlation between glycomic and transcriptomic changes in cells by analyzing the expression of *MGAT3* and *MGAT5*, which are responsible for the production of bisecting GlcNAcs and branched β1,6 GlcNAcs, respectively. *MGAT3* was specifically and considerably up-regulated (~16-fold) by the introduction of H-RasV12, which is completely consistent with our glycomic observations (Fig 4l, left). *MGAT5* was markedly up-regulated (~4-fold) by the introduction of hTERT, which is also consistent with the glycomic profile of tetra-antennary glycans (Fig 4l, right).

### Alterations in O-glycomics

#### Introduction of H-RasV12 suppresses the biosynthetic pathway of core 2 O-glycans

**Fig 5:**
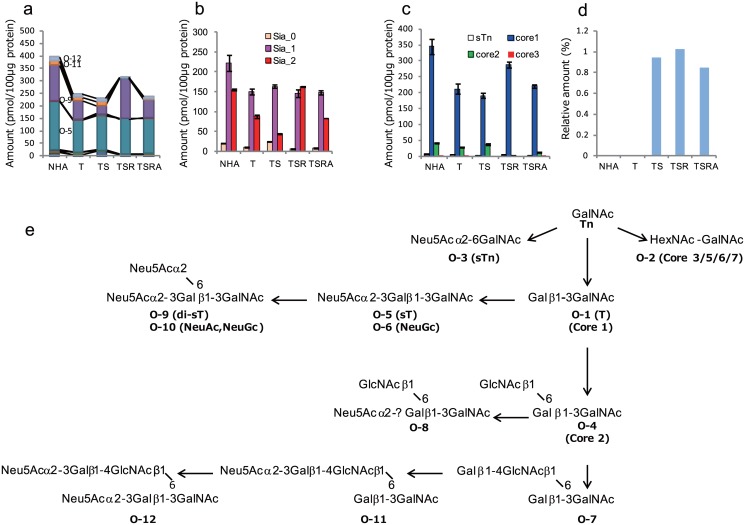
Cellular O-glycomes. Quantified O-glycans were classified according to their composition (a), number of sialic acid (Sia) residues (b), and core glycan structures (c). Comparison of relative levels of O-glycans showing N-glycolylneuraminic acid (Neu5Gc) incorporation among total sialylated O-glycans (d). Estimated biosynthetic pathway of O-glycans in NHA cells (e). Glycan classifications were based on the estimated glycan structures and compositions shown in S3 Table. Each value shown in b and d represents the mean ± range of two independent MALDI-TOF MS analyses.

In total, 12 O-glycans were quantitatively analyzed (S11–S15 Tables; S11 Fig). The fluctuations in total O-glycan expression levels were rather moderate following serial transformation of parental NHA cells, but various qualitative alterations were observed (Fig 5a). For example, upon introduction of SV40ER, the expression level of neutral O-glycans increased, while that of di-sialylated O-glycans decreased (Fig 5b). Subsequent introduction of H-RasV12 reduced the expression of neutral O-glycans and markedly increased the expression of di-sialylated O-glycans. Fucosylated O-glycans were below the limit of detection in all types of NHA cells.

To distinguish between core 2 and extended core 1 O-glycans, we performed enzymatic digestion of the cells using exo-β-galactosidase, followed by matrix-assisted laser desorption/ionization time-of-flight mass spectrometry (MALDI-TOF MS). Signals corresponding to (Hex)_2_(HexNAc)_2_(Neu5Ac)_1_ almost disappeared in NHA, NHA/TS, and NHA/TSR cells after enzymatic treatment, indicating that the branched structure (core 2) is more likely than the linear structure (extended core 1) (Fig 5e and S12 Fig). As shown in Fig 5c, core 2 O-glycan levels were markedly reduced upon introduction of H-RasV12.

#### Incorporation of Neu5Gc into O-glycans is increased after the introduction of SV40ER

Initially, no Neu5Gc-containing O-glycans were detected in NHA or NHA/T cells, but they were revealed after the introduction of SV40ER. O-glycans carrying Neu5Gc comprised ~0.8–1% of the total population of sialylated O-glycans (Fig 5d).

### Cluster analysis

**Fig 6:**
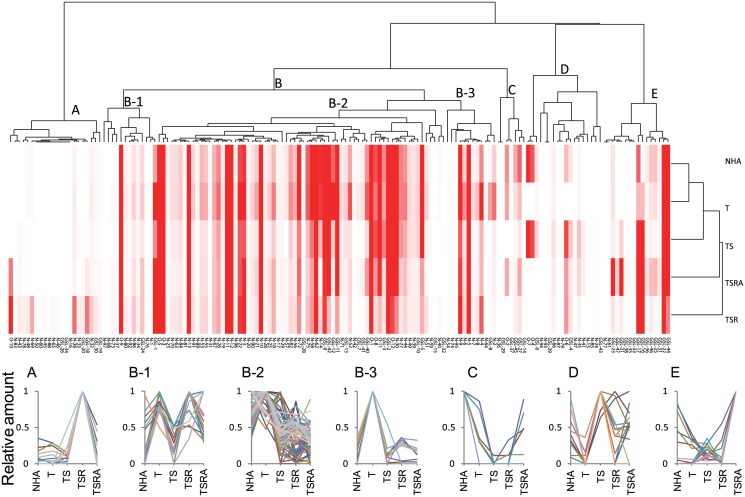
Classification of cells and glycans by N-, O- and GSL-glycomics based on unsupervised cluster analysis. The absolute amount of each glycan (pmol/100 μg protein) was analyzed by using Cluster 3.0 software. The heat map with clustering was acquired by using Java Threeview software. The relative abundance of each glycan (classified into Group A, B-1, B-2, B-3, C, D or E) is shown in the lower panel.

Hierarchical clustering analysis based on quantitative glycomic profiles of N-, O- and GSL-glycans yielded plausible classification of model cells (Fig 6). The greatest similarity was observed between NHA and NHA/T cells. The location of the NHA/TS cells was closest to that of the NHA and NHA/T cells, whereas the more transformed NHA/TSR and NHA/TSRA cells fell into distinct groups. The cells could be broadly classified into five categories based on relative amounts of up-regulated glycan expression, as follows: A) NHA/TSR, B) NHA/T, C) NHA, D) NHA/TS and E) NHA/TSRA cells. Group B (NHA/T cells) could be further subdivided into Groups B-1, B-2 and B-3. The cluster analysis indicated that many of the glycomic alterations occurring during glioma progression were temporal.

## Discussion

Since the early 1980s, introduction of representative oncogenes such as Src [33], Ras [34], and fps/fes [35], as well as oncogenic viruses such as the polyoma virus [36] were found to alter N-glycosylation profiles (leading to, for example, increased β1,6 GlcNAc branching, extension with polyLacNAc). However, ectopic expression of oncogenes alone in primary human cells is not sufficient to cause tumorigenic transformation and prior immortalization renders a variety of primary human cell types sensitive to Ras-mediated growth transformation [18]. The cancer-associated alterations in signal transduction pathways may lead to increased expression of specific glycosyltransferases, resulting in modified glycosylation patterns, while cancer-associated glycans expressed on cell membrane receptors can amend cell signaling, resulting in modulation of the basic properties of cancer cells [6]. However, the cause-versus-effect relationship between tumor progression and glycosylation changes remains mostly unclear.

**Fig 7:**
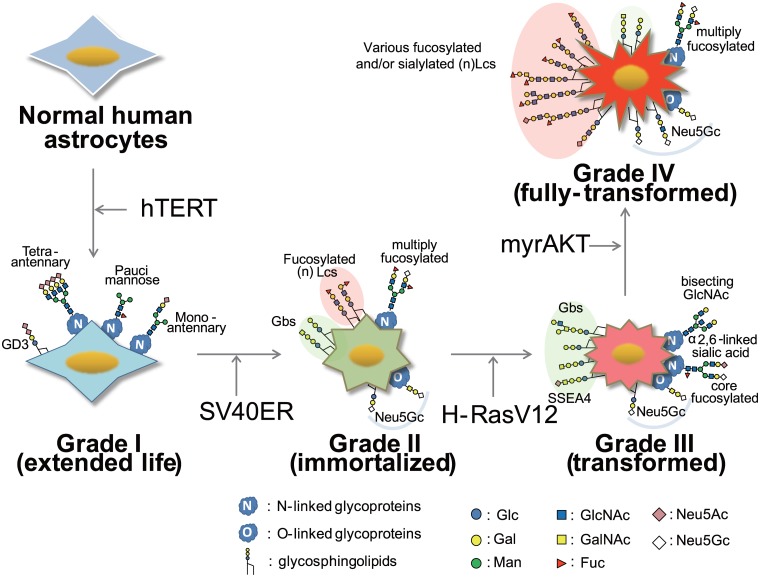
Representative glycans expressed at high levels upon ectopic expression of the various genes.

In this study, we undertook a comprehensive glycomic approach to overview the causal relationships between various phases of multistep tumorigenesis and glycosylation status by using a human brain tumor/glioma progression model. We are aware of the limits of this model, which reduces a complex disease in vivo to several genetic alterations in vitro. Nevertheless, the present investigation enabled a priori identification of a number of previously reported glycomic alterations during glioma advance, demonstrating the usefulness of the glioma progression model employed. As summarized in Fig 7 and Table 1, our study uniquely dissects the contribution of each tumorigenesis step to the glycosylation changes detected in the transformed astrocytes.

**Table 1:** Summary of the causal relationship between multistep tumorigenesis and glycomic alterations.

Observed phenomena	TERT	SV40ER	H-RasV12	MyrAKT	(Possible) Causal gene(s)
Total N-glycans	↑	↓↓	→	→	various
Pauci-mannose type N-glycans	↑↑	↓↓	↑	↓	unidentified
N-glycans with multiple fucose	→	↑↑	↓↓	↑	various FUTs
N-glycans with bisect GlcNAc	→	→	↑↑↑	↓↓↓	*MGAT3*
N-glycans with β1–6 linked GlcNAc	↑	↓	→	↓	*MGAT5*
N-glycans with β1–6 linked GlcNAc (relative abundance in total C/H glycans)	→	↑	→	→	*MGAT5* etc
N-glycans with monoantennary glycans	↑↑	↓↓↓	↑	→	*MGAT2*
Proportion of α2,6 sialic acids of total sialic acids in A1 and A2	↓	→	↑	↓	*ST6GAL I*, *II*, *ST3GAL III*, *IV*
Proportion of α2,6 sialic acids of total sialic acids in A1F and A2F	↓↓	↓↓↓	↑↑↑	↓↓↓	*ST6GAL I*, *II*, *ST3GAL III*, *IV*
N-glycans with Neu5Gc (%)	↑↑↑	↑	↑	↓	lysosomal sialic acid transporter/ lysosomal sialidase
**Total neutral O-glycans**	↓↓	↑↑	↓↓	↑	various SIATs
Disialylated O-glycans (mostly disialyl T)	↓	↓↓	↑↑	↓	*ST6GALNAC*
O-glycans with core2	↓	→	↓↓↓	↑↑	*B3GNT3* or *GCNT1*, *3*, *4*
O-glycans with Neu5Gc	→	↑↑↑	→	→	lysosomal sialic acid transporter/ lysosomal sialidase
Total GSL glycans	→	↓↓	↓	↑↑	various
LacCer	↑↑↑	↓↓↓	↑↑↑	↓↓	*UGCG/B4GALT6*
Total ganglio-series GSL glycans	→	↓↓	↓↓↓	↑	balance of *ST3GAL5*, *B4GALNT1*, *A4GALT* and *B3GNT5*
GM3	↓	→	↓↓	↑	*ST3GAL5*
GM2	↓	↓	↓↓↓	↑	*B4GALNT1*
GM1	→	↓↓	↓↓	→	*B3GALT4*
GD3	↑↑↑	↓↓↓	↑	→	*SIAT8A*
GD1	↑	↓↓	↓↓↓	↑↑	*B3GALT4*
Total globo-series GSL glycans	↓	↑↑↑	↑↑	↑↑	balance of *ST3GAL5*, *B4GALNT1*, *A4GALT* and *B3GNT5*
Gb3	↑↑↑	↑↑↑	↑↑	↑	*A4GAT*
Gb4	↓↓	↑↑↑	↑↑	↑	*B3GALNT1*
SSEA-4	→	↓	↑↑↑	↓	*ST8SIA1*
Galβ1-4GlcNAcβ1-6(Galβ1–3)GalNAcβ1-3Galα1-4Galβ1-4Glc	→	↑↑↑	↑↑↑	↓↓↓	GNTs and GALTs
Total lacto-series GSL glycans	→	↓↓	→	↑↑↑	balance of *ST3GAL5*, *B4GALNT1*, *A4GALT* and *B3GNT5*
Lc-series glycans with fucose	↓↓↓	↑↑↑	↓↓	↑↑↑	various FUTs
Galα1-3Galβ1-4GlcNAcβ1-3Galβ1-4GlcNAcβ1-3Galβ1-4Glc	→	↓↓	↑↑↑	↓↓	GNTs and GALTs
GSL glycans with sialic acid(s)	→	↓↓	↓↓	↑↑↑	various SIATs
GSL glycans with Neu5Gc	→	↑↑	↑	→	balance of *ST3GAL5*, *B4GALNT1*, *A4GALT* and *B3GNT5*

Increase by 1.25–2-fold (↑), 2–5-fold (↑↑), and > 5-fold (↑↑↑). Decrease by 0.5–0.75-fold (↓), 0.2–0.5-fold (↓↓), and < 0.2-fold (↓↓↓). The increase and decrease is within 0.75-fold and 1.25-fold (→)

### Effects of hTERT

The expression levels of N-glycans with a β1,6-branched structure, as well as the expression level of *MGAT5*, were maximized by the introduction of hTERT, which is reasonable considering that *MGAT5* is involved in cancer development [13]. Other N-glycosylation changes included the augmented expression of PM- and mono-antennary type glycans. PM-type glycans were previously thought to be expressed primarily by plants and invertebrates, and absent or only weakly expressed in normal vertebrate tissue. Although PM-type glycans are synthesized in *Caenorhabditis elegans* through the actions of α-3,6-mannosidase II and β-N-acetyl-hexosaminidase [37], the latter is not found in vertebrates and the mechanism for the production of PM in vertebrates is unknown. It should be noted that PM-and mono-antennary type glycans may be generated from the same precursor (Gn-M3) (S13 Fig).

We also observed a specific increase in GD3 expression upon the introduction of hTERT, consistent with the crucial regulatory roles that GD3 plays in normal physiological processes (e.g., embryogenesis and brain development [38]) and under pathological conditions (e.g., tumor onset and progression [39]). Recently, mutation of the hTERT promoter was documented during the early stages of gliomagenesis [40], suggesting that these glycomic alterations are candidates for the early diagnosis of gliomas.

### Effects of hTERT and SV40ER

In addition to hTERT actions, SV40ER-mediated inactivation of the p53 and Rb tumor suppressors is central to human cell immortalization. Here, introduction of SV40ER into NHAs reduced the total levels of N- and GSL-glycans to less than one-half. Nonetheless, a marked increase in the expression of multiply fucosylated complex N-glycans was noted. Gene expression analysis of major FUTs (*FUT1-9*) could not explain the elevated levels of multiply fucosylated N-glycans upon SV40ER introduction, indicating a complex regulation between glycogene transcription and glycan presence. Various fucosylated (n)Lc-series glycans also appeared in astrocytes after the introduction of SV40ER. Introduction of SV40ER likewise caused a relative increase in the expression of neutral glycans and a decrease in sialylated glycans, regardless of the type of glycoconjugate (N-, O-, or GSL-glycans; S14 Fig) present, which may increase the binding capacity of galectins whose importance in cancer is increasingly recognized [6]. Core structural changes from Gg- to Gb-series glycans during astrocyte immortalization were also found, where the Gg- to Gb-series transition represents a reciprocal change in the Gb- to Gg-series switch observed upon human embryonic stem cell differentiation into the embryoid body [41].

### Effects of hTERT, SV40ER, and Ras

Activation of Ras together with hTERT and SV40ER induced the expression of bisecting GlcNAc-containing N-glycans in astrocytes. Interestingly, co-expression of H-RasV12 and myrAKT reduced the levels of N-glycans bearing bisecting GlcNAc to levels similar to those seen in NHA-TS cells. In other words, the expression of bisecting GlcNAc decreased in spite of the increased malignancy imparted by AKT. This finding is consistent with our previous observations indicating that tumor foci with low levels of cell proliferation indices as Ki67, preferentially express glycans with bisecting GlcNAc residues [42].

The function of bisecting GlcNAc in glioma progression is still unknown. However, during gastric carcinoma formation, E-cadherin and integrins alter their glycosylation status by promoting *MGAT3* expression during gastric carcinoma formation. *MGAT3* increases intercellular adhesions and down-regulates intracellular signaling pathways involved in cell motility [43, 44]. Here, H-RasV12 introduction also prompted a transient increase in α2,6-sialylation. Increased adhesion of ST6Gal I-transfected cells to extracellular matrix (ECM) substrates (e.g., fibronectin, laminin, and collagen) was previously reported in colon cancer [45]. The observation that two characteristic and transient N-glycomic alterations function to increase cell-cell and cell-ECM adhesions suggests their roles to promote tumor progression via interactions with adjacent cells and the ECM. This hypothesis supports findings that bisecting GlcNAc can promote cancer growth under some circumstances [46, 47].

Ras activation additionally led to increased expression of *FUT8*, which agrees with a previous report showing strong LCA-lectin binding in tissue glioma cells, whereas the LCA binds to sugar chains containing core fucose [14]. It may be worth mentioning that the presence of core fucose reportedly enhances affinity toward galectin-3 [48], a pleiotropic carbohydrate-binding protein involved in various normal and pathological biological processes, including cancer progression and metastasis [49, 50]. The presence of core fucose has been reported to have a dramatic effect on the conformation of the Manα1–6 antenna, resulting in reduced antenna flexibility [51].

H-RasV12 introduction suppressed the biosynthetic pathway of core 2 O-glycans in NHA cells. The attenuated cellular ability to synthesize O-glycans might result in the accumulation of T-antigen (Gal β1-3GalNAc), followed by the thorough sialylation of T-antigen to produce disialyl T-antigen (Fig 5c). The characteristic switch from core 2 structures to T-antigen accumulation was recently documented during neoplastic transformation of the breast epithelium [52], which may also be attributable to the activation of Ras. In addition, the introduction of H-RasV12 resulted in a prominent decrease in Gg-series GSLs, a concomitant increase in Gb-series glycans, a specific increase in the recently reported glioma marker, SSEA-4, as well as an increase in a Gb-series glycan, Galβ1,4GlcNAcβ1,6(Galβ1,3)GalNAcβ1,3Galα1,4Galβ1,4Glc. The latter may be worthwhile for further evaluation as a novel glioma biomarker.

### Effects of hTERT, SV40ER, Ras, and AKT

Multiply fucosylated N-glycan content markedly increased upon activation of AKT. Observed up-regulations of *FUT1*, *FUT3* and *FUT5* upon introduction of myrAKT suggest the increased expression of Lewis B and/or Lewis Y epitopes. Notably, increased expression of those epitopes has been reported in various cancers [53]. Because fucosylation regulates the activity of several growth factors, and growth factor/ cytokine receptors (e.g., epidermal growth factor receptor and transforming growth factor β receptor), an increase in fucosylated N-glycans might positively contribute to the full-transformation phenotype induced herein by myrAKT. Cancer cell invasiveness, regulated by the interaction between fibronectin and α5β1 integrin, is similarly affected by fucosylation [1].

The most characteristic alteration upon introduction of myrAKT into NHA cells may be the striking increase in the expression levels of (n)Lc-series glycans, including extended and/or branched polyLacNAc structures bearing sialic acid/fucose modification(s). Like the N-glycans, elevated levels of fucosylated glycans were also discerned following myrAKT presentation, with the increased levels especially-pronounced for the (n)Lc-series GSL-glycans. Because no fucosylated O-glycans were detected, the level of fucosylation apparently differed depending on the type of glycoconjugate.

Because human cells lack the enzyme required to synthesize Neu5Gc (CMP-Neu5Ac hydroxylase), glycans containing Neu5Gc are usually absent in normal human tissues/cells. However, trace amounts of Neu5Gc can be taken up by and incorporated into human cells (especially cancer cells and stem cells) from external sources, such as dietary animal-based foods and FBS in the culture medium [54]. In this study, we observed that Neu5Gc incorporation into various glycoconjugates was markedly increased upon introduction of SV40ER, and that the relative levels of Neu5Gc versus Neu5Ac were lowest in N-glycans (0.02–0.05%), higher in O-glycans (~1%), and highest in GSL-glycans (2–4.5%). To the best of our knowledge, no other studies have reported differences in the incorporation efficiency of Neu5Gc among different types of glycoconjugates. Since CMP-Neu5Ac is the substrate for the hydroxylase, the acceptor- specificity of sialyltransferases may be responsible for this uneven distribution of Neu5Gc. Separate CMP-Neu5Ac transporters for different Golgi compartments may account for the differences in Neu5Gc incorporation efficiencies among the different types of glycoconjugates. Nonetheless, the mechanism underlying differential cell-type specific incorporation efficiencies is a subject for further exploration.

Given that glycomic profiling transitions take place throughout the transformation process of NHA cells, epigenetic regulation may contribute to the specific expression of glycosylation-related enzymes. In fact, Kizuka and colleagues reported epigenetic regulation of brain-specific glycosyltransferases, where histone deacetylase (HDAC) 11 silenced N-acetylglucosaminyltransferase IX (GnT-IX) and also activated the GnT-IX promoter by complex formation with O-GlcNAc transferase and ten-eleven translocation-3 (TET3) [55].

In conclusion, the current study highlights the importance of a systematic global overview of the glycomics of model tumor cells with defined genetic elements to our understanding of how deregulated cellular pathways can affect total cellular glycosylation. The current study demonstrates that glycomic alterations during the transformation process are quite dynamic and transient rather than static and enduring. Furthermore, we found that similar glycan expression profiles among different stages of transformation could be caused by distinct genetic alterations, and that combinations of plural genetic alterations could offset the glycomic alterations caused by one genetic alteration. This may explain, to some extent, the differential interpretation of impaired glycan expression by different investigators (e.g., positive and negative effects of bisecting GlcNAc on cancer cell growth). Although the currently employed multistep model provides just one prospective scenario underlying tumorigenesis in the human astrocyte, extending the same approach to other models is expected to lead to a better understanding of systems glycobiology, both during homeostasis and over the course of disease progression.

Finally, it should be noted that all glycans showing altered expression during immortalization and transformation are biomarker candidates for glioma diagnosis. Studies aiming to identify biomarkers for brain tumors through the use of pathological tissue specimens are now in progress. We anticipate that the significance of many of the glycomic alterations observed in this study will be unveiled by clinical research in the near future.

## Supporting Information

S1 Fig Reproducibility of the BEP-based method employed in the current study for the analysis of O-glycans. Each value is shown as the mean ± the SD of three independent MS analyses.
(TIF)

S2 Fig Representative MS spectra showing the GSL-glycomic profiles of NHA, NHA/T, NHA/TS, NHA/TSR and NHA/TSRA. Composition of the numbered oligosaccharide signals are summarized in S1 Table.
(EPS)

S3 Fig Expression profiles of (n)Lc-series GSL-glycans. (a) Expression profiles of all (n)Lc-series GSLglycans detected in this study. (b) Fucosylation status of (n)LC-series GSL-glycans. (n)Lc-glycans were quantified in NHA, NHA/T, NHA/TS, NHA/TSR, and NHA/TSRA cells and classified according to the number of fucose residues. (c) Expression profiles of representative fucosecontaining (n)Lc-series glycans. SphinGOMAP (http://www.sphingomap.org/) online databases were used for structural estimation. Each value represents the mean ± SD of three independent MALDI-TOF MS analyses.
(EPS)

S4 Fig Biosynthetic pathways of lacto-series GSL-glycans in NHA, NHA/T, NHA/TS, NHA/TSR, and NHA/TSRA cells. SphinGOMAP (http://www.sphingomap.org/) online databases were used for structural estimation, and all possible isomers are shown.
(EPS)

S5 Fig Representative MS spectra showing the N-glycomic profiles of NHA, NHA/T, NHA/TS, NHA/TSR and NHA/TSRA. Composition of the numbered oligosaccharide signals are summarized in S2 Table.
(EPS)

S6. Fig Expression profiles of each N-glycan bearing multiple fucose residues. Each value represents the mean ± SD of three independent MALDI-TOF MS analyses.
(EPS)

S7 Fig Real-time PCR analysis of FUT1-9 mRNA expression levels. Each value represents the mean ± the standard deviation (SD) of three independent real-time PCR analyses.
(TIF)

S8 Fig Effect of α2,3-neuraminidase on the amounts of di-sialyl, mono-sialyl, and asialylated biantennary N-glycans. Upper panel: A2, A1, and NA2 glycans. Lower panel: A2F, A1F, and NA2F glycans.
(EPS)

S9 Fig Branching status of N-glycans. N-Glycans were quantified in NHA/T, NHA/TS, NHA/TSR, and NHA/TSRA cells and classified according to the number of HexNAc residue(s). Each value represents the mean ± SD of three independent MALDI-TOF MS analyses.
(EPS)

S10 Fig Expression profiles of each N-glycan bearing a bisecting GlcNAc residue. Each value represents the mean ± SD of three independent MALDI-TOF MS analyses.
(EPS)

S11 Fig Representative MS spectra showing the O-glycomic profiles of NHA, NHA/T, NHA/TS, NHA/TSR and NHA/TSRA. Composition of the numbered oligosaccharide signals are summarized in S3 Table.
(EPS)

S12 Fig Biosynthetic pathways of extended core 1-type and core 2-type O-glycans (a) and MS spectra showing the effect of β-galactosidase, which was used to discriminate between extended core a-type and core 2-type O-glycans. (EPS)

S13 Fig Estimated biosynthetic pathways of pauci-mannose and mono-antennary-type N-glycans. (EPS)

S14 Fig Relative abundances of neutral N-, O- and GSL-glycans in NHA, NHA/T, NHA/TS, NHA/TSR and NHA/TSRA cells. (EPS)

S1 Table List of GSL glycans detected in NHA. (XLSX)

S2 Table List of GSL glycans detected in NHA/T. (XLSX)

S3 Table List of GSL glycans detected in NHA/TS. (XLSX)

S4 Table List of GSL glycans detected in NHA/TSR. (XLSX)

S5 Table List of GSL glycans detected in NHA/TSRA. (XLSX)

S6 Table List of N-glycans detected in NHA. (XLSX)

S7 Table List of N-glycans detected in NHA/T. (XLSX)

S8 Table List of N-glycans detected in NHA/TS. (XLSX)

S9 Table List of N-glycans detected in NHA/TSR. (XLSX)

S10 Table List of N-glycans detected in NHA/TSRA. (XLSX)

S11 Table List of O-glycans detected in NHA. (XLSX)

S12 Table List of O-glycans detected in NHA/T. (XLSX)

S13 Table List of O-glycans detected in NHA/TS. (XLSX)

S14 Table List of O-glycans detected in NHA/TSR. (XLSX)

S15 Table List of O-glycans detected in NHA/TSRA. (XLSX)
