# Supplementary figures and images for: Comprehensive Glycomics of a Multistep Human Brain Tumor Model Reveals Specific Glycosylation Patterns Related to Malignancy
Source: PLoS One. 2015 Jul 1;10(7):e0128300. doi: 10.1371/journal.pone.0128300 (PMC4488535; doi:10.1371/journal.pone.0128300)

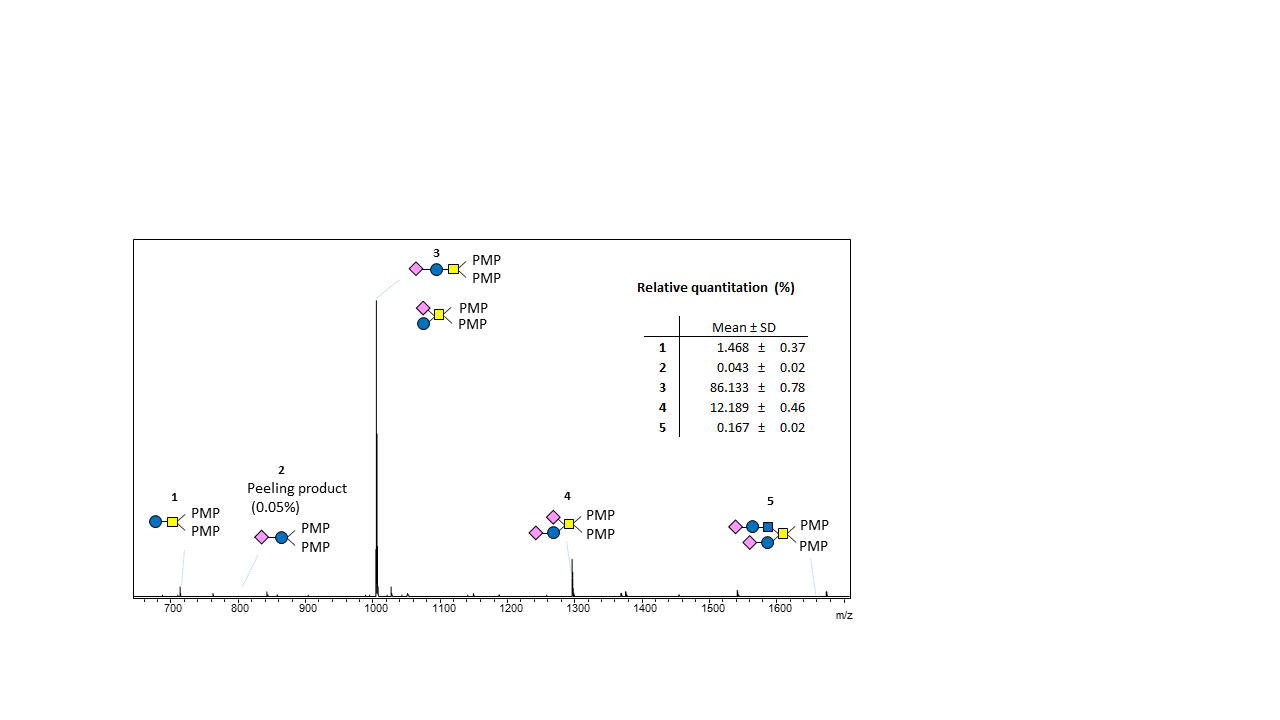

Supplement: S1 Fig — Each value is shown as the mean ± the SD of three independent MS analyses. (TIF) [file pone.0128300.s001.tif]

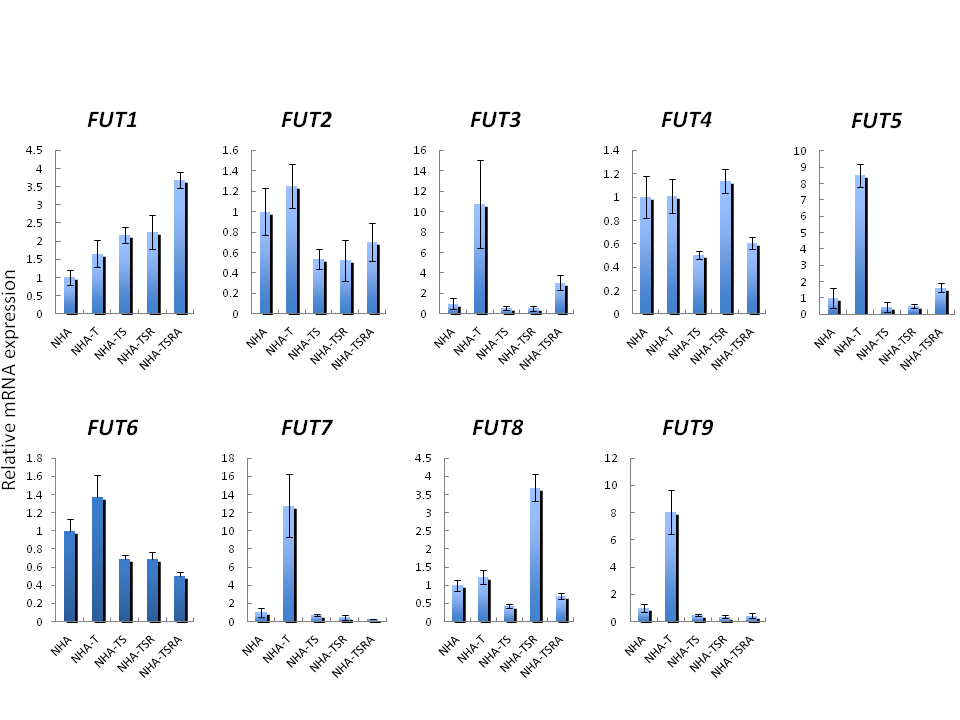

Supplement: S7 Fig — Each value represents the mean ± the standard deviation (SD) of three independent real-time PCR analyses. (TIF) [file pone.0128300.s007.tif]
